# Testosterone Induces Molecular Changes in Dopamine Signaling Pathway Molecules in the Adolescent Male Rat Nigrostriatal Pathway

**DOI:** 10.1371/journal.pone.0091151

**Published:** 2014-03-11

**Authors:** Tertia D. Purves-Tyson, Samantha J. Owens, Kay L. Double, Reena Desai, David J. Handelsman, Cynthia Shannon Weickert

**Affiliations:** 1 Schizophrenia Research Institute, Sydney, New South Wales, Australia; 2 Schizophrenia Research Laboratory, Neuroscience Research Australia, Sydney, New South Wales, Australia; 3 Discipline of Biomedical Science, School of Medical Sciences, Sydney Medical School, University of Sydney, Sydney, New South Wales, Australia; 4 School of Medical Sciences, University of New South Wales, Sydney, New South Wales, Australia; 5 ANZAC Research Institute, University of Sydney, Concord Hospital, Concord, New South Wales, Australia; 6 School of Psychiatry, University of New South Wales, Sydney, New South Wales, Australia; Prince Henry's Institute, Australia

## Abstract

Adolescent males have an increased risk of developing schizophrenia, implicating testosterone in the precipitation of dopamine-related psychopathology. Evidence from adult rodent brain indicates that testosterone can modulate nigrostriatal dopamine. However, studies are required to understand the role testosterone plays in maturation of dopamine pathways during adolescence and to elucidate the molecular mechanism(s) by which testosterone exerts its effects. We hypothesized that molecular indices of dopamine neurotransmission [synthesis (tyrosine hydroxylase), breakdown (catechol-*O*-methyl transferase; monoamine oxygenase), transport [vesicular monoamine transporter (VMAT), dopamine transporter (DAT)] and receptors (DRD1-D5)] would be changed by testosterone or its metabolites, dihydrotestosterone and 17β-estradiol, in the nigrostriatal pathway of adolescent male rats. We found that testosterone and dihydrotestosterone increased DAT and VMAT mRNAs in the substantia nigra and that testosterone increased DAT protein at the region of the cell bodies, but not in target regions in the striatum. Dopamine receptor D2 mRNA was increased and D3 mRNA was decreased in substantia nigra and/or striatum by androgens. These data suggest that increased testosterone at adolescence may change dopamine responsivity of the nigrostriatal pathway by modulating, at a molecular level, the capacity of neurons to transport and respond to dopamine. Further, dopamine turnover was increased in the dorsal striatum following gonadectomy and this was prevented by testosterone replacement. Gene expression changes in the dopaminergic cell body region may serve to modulate both dendritic dopamine feedback inhibition and reuptake in the dopaminergic somatodendritic field as well as dopamine release and re-uptake dynamics at the presynaptic terminals in the striatum. These testosterone-induced changes of molecular indices of dopamine neurotransmission in males are primarily androgen receptor-driven events as estradiol had minimal effect. We conclude that nigrostriatal responsivity to dopamine may be modulated by testosterone acting via androgen receptors to alter gene expression of molecules involved in dopamine signaling during adolescence.

## Introduction

Schizophrenia is slightly more common in males than females [1]. The peak age of onset of schizophrenia in males is concomitant with higher testosterone levels at adolescence and young adulthood, suggesting testosterone may be linked to the onset of psychosis in vulnerable individuals [2]. Increased dopamine within the nigrostriatal pathway of patients with schizophrenia is proposed as a driver of psychosis [3]–[5] supported by the effectiveness of antipsychotics (which block dopamine D2 receptors) in diminishing symptoms of hallucinations and delusions [6]. Imaging studies provide direct evidence of dysregulation of striatal dopamine transmission in schizophrenia underlying the development of psychosis [7], [8] but the underlying molecular cause(s) of this dopamine dysregulation are unknown. Understanding the molecular mechanisms by which testosterone modulates the maturation and regulation of nigrostriatal dopamine responsivity during adolescence is crucial to understanding the possible role of testosterone in schizophrenia risk.

Dopaminergic transmission involves signaling by five G-protein coupled receptors divided into inhibitory receptors (DRD2, DRD3, DRD4) and excitatory receptors (DRD1, DRD5), as well as the regulation of dopamine movement across membranes via dopamine transporter (DAT) and vesicular monoamine transporter (VMAT2). Dopamine is broken down by catechol-O-methyl transferase (COMT) and monoamine oxidase (MAOA and MAOB) enzymes to the main metabolites, 3,4-dihydroxyphenylacetic acid (DOPAC) and homovanillic acid (HVA) (reviewed in [9]). Dopamine homeostasis is maintained by dopamine biosynthesis, transport and breakdown, all potentially modulated by testosterone-induced changes in gene expression and/or protein levels of the molecules involved.

In order for testosterone to change gene expression, it can bind directly to the transcription factors, androgen receptor (AR) or, following conversion to estradiol by aromatase, to estrogen receptors (ERα, ERβ). Testosterone is also converted to dihydrotestosterone (DHT), a more potent, pure androgen which initiates transcription mainly via ARs [10] (although it can also act through ERβ [11]). All three receptors are expressed in dopaminergic neurons in the adult rodent midbrain [12]–[15] and ERα and AR expression has been confirmed in the adolescent male rat midbrain [16].

Previously, we found that testosterone increased TH in the adolescent substantia nigra [16]; and here, we predicted that activation of sex steroid receptors in male adolescence would lead to increased striatal TH protein and dopamine. Evidence regarding the mechanism(s) by which testosterone modulates nigrostriatal dopamine neurotransmission in the adult mammalian brain is, however, conflicting [17]–[21]. Circulating testosterone levels are positively correlated with protein levels of tyrosine hydroxylase (TH), the rate-limiting enzyme in dopamine synthesis, in the striatum of adolescent male rhesus macaques [22]. In addition, gonadectomy reduced TH activity in the striatum of adult male rats and this reduction was prevented by testosterone [23].We recently reported that testosterone increased COMT and MAO mRNAs in the adolescent male rat substantia nigra (SN), implying increased dopamine turnover capacity in the nigrostriatal pathway in male adolescence [16]. Further studies in adult rats suggest other components of dopamine signaling can also be modified by androgens [6], [24]–[26]. In the current work, we tested the hypothesis that testosterone can induce androgen receptor-driven changes in gene expression of multiple molecules involved in the regulation of dopamine neurotransmission in the nigrostriatal pathway in adolescent male rat brain.

In the current work, we investigated which molecular indices of dopamine neurotransmission are modulated by testosterone and whether testosterone actions in the adolescent male rat nigrostriatal pathway are driven primarily by androgenic or estrogenic mechanisms. We achieved this by gonadectomizing 45-day old male rats (adolescence) and replacing endogenous testosterone with either testosterone (T), DHT or 17β-estradiol (estradiol, E) until 60 days of age (young adults). Unexpectedly, dopamine was unchanged in the dorsal striatum by gonadectomy or with testosterone replacement, but dopamine turnover was increased by gonadectomy. Gene expression for dopamine transporters (DAT and VMAT2) and protein levels of DAT were increased in the substantia nigra by androgens but not estradiol. DAT protein levels were unchanged in the striatum. We show that, in contrast to our previously reported data in the substantia nigra [16], striatal COMT and MAO gene expression is unchanged by sex steroids. We report distinct dopamine receptor (D1, D2, D3, D5) mRNAs are modulated in areas of dopaminergic cell bodies and post-synaptic targets (D2 and D5) of the nigrostriatal pathway, mostly via AR activation. Our results indicate that in adolescent male rats, testosterone may alter the dopamine neurotransmission within the nigrostriatal pathway by attenuating dopamine turnover in the striatum and by modulating mRNA levels of dopamine receptors and changing dopamine transporter levels in the midbrain.

## Materials and Methods

### Experimental animals

All animal experiments were approved by the Animal Care and Ethics Committee of the University of New South Wales (Ethics number ACEC10/40) in accordance with the National Health and Medical Research Council of Australia's Code of Practice for the Care and Use of Animals for Experimental Purposes, which also conforms to standard international guidelines. Male Sprague-Dawley rats were used for all experiments (Animal Resource Centre, Perth, WA, Australia). Rats were group housed (3–4/cage) in 12/12 hr light/dark phases with constant humidity and temperature and free access to water and standard rat chow. All surgery was performed under ketamine hydrochloride and xylazine hydrochloride anaesthesia, and all efforts were made to minimize suffering. A total of 78 experimental animals were used.

#### Gonadectomy and sex steroid replacement

Male rats were gonadectomised at 45 days of age and given continuous replacement testosterone (T), DHT or 17β-estradiol (E) by subdermal silastic implant [27]–[29] for two weeks. Male Sprague-Dawley rats experience an increase in circulating testosterone between 45 and 60 days of age [30]–[34] and at 60 days of age are considered young adults. There were five groups (∼15 rats/group): intact (Intact); gonadectomy alone (Gdx); gonadectomy plus testosterone (Gdx+T); gonadectomy plus DHT (Gdx+DHT); gonadectomy plus estradiol (Gdx+E). Rats were anaesthetized with an intraperitoneal injection of ketamine hydrochloride (60 mg/kg) and xylazine hydrochloride (10 mg/kg) (Provet, Castle Hill, Australia). Intact animals underwent abdominal surgery but gonads were left in place. Silastic implants were placed subcutaneously between the shoulder blades at time of gonadectomy. Gdx and Intact groups were given empty implants. Implants were 1 cm long, internal diameter 1.47 mm, outer diameter 1.95 mm and filled with crystalline steroid (ends sealed with silastic adhesive). The implants have been characterized in previous studies and achieve supraphysiological, steady-state serum hormone levels and androgen implants maintain seminal vesicle weights comparable to that in untreated animals [16], [28], [35]. T, DHT and E were quantified in serum using stable isotope dilution liquid chromatography-tandem mass spectroscopy as described [36] and adapted for rodents [37]. The limit of quantitation is 0.025 ng/ml T, 0.1 ng/ml DHT and 5 pg/ml E as previously reported [16]. Concentrations of circulating sex steroids and seminal vesicle weights confirm that successful sex steroid replacement was achieved in these rats as reported previously [16]. Briefly, in Intact rats circulating concentrations of T and DHT were 2.8 ± 0.6 and 0.2 ± 0.03 ng/ml respectively and E was 7.3 pg/ml. In Gdx rats, T was 0.03 ± 0.001 ng/ml and DHT and E were not detectable. In Gdx+T rats, T was 23.1 ± 12.0 ng/ml and DHT and E were not detectable. In the Gdx+DHT group, DHT was 21.42 ± 10.6 ng/ml and T was not detectable. In the Gdx+E group, E was 17.0 ± 6.7 pg/ml.

#### Brain dissection

At 60 days of age, rats were anaesthetized with 60 mg/kg sodium pentobarbital (Euthal, Delvet, Seven Hills, Australia) and decapitated. Brains were removed and tissue blocks containing midbrain and striatum were dissected following a Rat Brain Atlas [38]. The midbrain block was trimmed transversely at the cerebral aqueduct and two lateral segments of midbrain on either side of the ventral tegmental area containing the substantia nigra (SN) were collected. The block containing striatum (between bregma 2.28 mm and 0.36 mm) was trimmed on either side of the midline along the lateral ventricles and ventrally to remove non-striatal tissue. In the striatal blocks, the striatum above the line of the anterior commissures was collected as dorsal striatum and referred to in the study as striatum. The striatum was separated from the overlying corpus callosum and stored. Trunk blood was collected on the day of euthanasia in 0.8 ml serum gel tubes (Z serum MiniCollect tube, GreinerBioOne, Wemmel, Belgium) and serum collected by centrifugation. Left and right hemisphere segments were randomly assigned for either protein or RNA extraction. 6–15 mg of tissue was separated from dorsal striatum segments allocated for RNA to utilize for reverse-phase, high pressure liquid chromatography.

### Dopamine and metabolite measurement

Dorsal striatum samples were analysed for dopamine, DOPAC and HVA, using high pressure liquid chromatography with electrochemical detection as previously described [39], [40]. Frozen tissue samples were sonicated (S-250A Sonicator; Branson Ultrasonics, Danbury, CT, USA) for 30 s in 19× the tissue weight of 150 mM H_3_PO_4_ and 500 µM diethylenetriaminepenta-acetic acid (Sigma, St Louis, MO, USA) followed by centrifugation (44,5000 g, 25 min, 4°C; Optima L-90K ultracentrifuge; Beckman Coulter, Palo Alto, CA, USA) and supernatant collected and stored (−80°C).

High pressure liquid chromatography was performed with a Prominence system (Shimadzu, Kyoto, Japan) composed of degasser (DGU-20A3), liquid chromatographer (LC-20AD), autosampler (SIL-20A) and communications module (CBM-20A). Samples were injected onto a Gemini C18 column (150×4.60 mm, 110 Å, 5 µm particle size; Phenomenex, Torrance, CA, USA) connected to an electrochemical detector (Antec Leyden Intro, Zoeterwoude, NV, Netherlands) with an Ag/AgCl reference electrode at a potential of +0.72 V and 40°C. Isocratic mobile phase contained 16% methanol, 84% 0.01 M monobasic sodium phosphate, 0.1 mM ethylenediaminetetraacetic acid, 0.65 mM 1-octane sulfonic acid, 0.5 mM triethylamine at pH 3.4, adjusted with hydrochloric acid (Sigma). Injection volumes were 20–35 µL with a 0.75 mL/min flow rate and a 23.5 min run time.

External standards (1 µM dopamine, 1 µM DOPAC and 2 µM HVA; Sigma) were run daily (9 days in total) to produce a six-point standard curve for dopamine (0.95–17.07 ng), DOPAC (0.84–15.13 ng) and HVA (1.82–32.79 ng) to quantify samples run on the same day. Peak area analysis was with Class VP7.3 SP1 software (Shimadzu). Aliquots of 20–35 µL of samples, pooled from a subset of thirty-five rats (seven samples from each group) were injected daily and used to normalise between measurements acquired on different days. Dopamine turnover was calculated as DOPAC+HVA/dopamine.

### Immunoblotting

Dorsal striatum or substantia nigra tissue blocks were homogenized (0.1 M Tris, pH 7.5, 50% glycerol, proteinase inhibitor cocktail (Sigma Cat# P8340) and aprotinin 0.015 mM, Sigma) using a handheld electric homogenizer (Polytron, Kinematica, Lucerne, Switzerland). Protein concentration was determined using the Bradford protein assay (Sigma). An aliquot of each sample was combined and used as a standard and run in duplicate on each gel to allow standardization between blots (internal control). Standard curves with between 0.5 and 20 µg substantia nigra or dorsal striatum protein were run and TH and DAT expression was determined to be within a linear range and 3 µg protein/sample was used. SDS-PAGE (10% acrylamide for TH and 8% acrylamide for DAT) was performed and proteins were transferred to nitrocellulose (45 µm, Biorad, CA, USA). Primary antibodies were anti-TH (host species rabbit, 1∶5000; Chemicon), anti-DAT (host species rabbit, 1∶300, H-20 sc14002, Santa Cruz) and anti-β-actin (host species mouse, 1∶5000; MAB1501, Millipore). Secondary antibodies were goat anti-mouse or anti-rabbit horseradish peroxidase conjugated (1∶4000, Millipore). Immunoreactive bands were detected using the LumiGlo detection kit (LumiGlo Reagent; Cell Signaling, Danvers, MA, USA) on hyperfilm (Amersham, GE Healthcare, Uppsala, Sweden). The immunoblots were scanned and band densities converted to numerical values using ImageJ software (ImageJ 1.43u, National Institutes of Health, USA). Relative intensities of TH and DAT bands (on separate blots) were normalized to relative intensity of β-actin bands on the same immunoblot.

### RNA extraction and cDNA synthesis

Total RNA was extracted from SN and dorsal striatum samples in 800–1000 µl TRIzol (Life Technologies, Grand Island, NY, USA) as recommended by the manufacturer. RNA was quantified using a ND-1000 spectrophotometer (Nanodrop Technologies, Wilmington, DE, USA) and RNA integrity was assessed with high resolution capillary electrophoresis (Agilent Bioanalyzer 2100, Agilent Technologies, Palo Alto, CA, USA). Two aliquots of 3 µg RNA from each sample were reverse transcribed with SuperScript III First-Strand Synthesis Supermix and random hexamers, according to the manufacturer's protocol (Life Technologies). The 2 aliquots of cDNA from each sample were pooled and diluted for qPCR. A parallel reaction was performed without reverse transcriptase.

### Quantitative real-time PCR (qPCR)

Transcripts of interest were measured by TaqMan Gene Expression Assays (Table 1) (Applied Biosystems, Foster City, CA, USA) using an ABI Prism 7900HT Fast Real-Time PCR System and a 384-well format. Three housekeeping genes were used to calculate the normalizing control for gene expression (termed geometric mean) and were selected on the basis that they were unchanged by the treatment. GusB, 18S rRNA and GAPDH, were used in the SN and GusB, GAPDH and YWHAZ were used in the striatum (Table 1, Gene names and Taqman probes). The geometric means of the three housekeeping genes used for each region were calculated as described previously [41]. Samples were run alongside a seven-point standard curve using serial dilutions of cDNA derived from SN or striatum RNA pooled from a subset of 25 rats (taken from all treatment groups). No template controls were included. Measurements were performed in triplicate. PCR cycling conditions were: 50°C for 2 min, 95°C for 10 min, 50 cycles of 95°C for 15 s and 60°C for 1 min. PCR data were captured with Sequence Detector Software (SDS version 2.4, Applied Biosystems) and real-time fluorescence intensity plotted with the threshold within the linear phase of the amplification profiles.

**Table 1:** Genes of interest and housekeeper genes with ABI Taqman Gene Expression Assay part numbers.

Gene symbol	Gene name	Taqman assay
**GUSB**	*Glucuronidase-β*	Rn00566655
**GAPDH**	*Glyceraldehyde 3-phosphate dehydrogenase*	Rn01775763
**18SRNA**	*18S ribosomal RNA*	Hs99999901
**YWHAZ**	*Tyrosine 3-monooxygenase*	Rn00755072
**DRD1**	*Dopamine receptor D1a*	Rn03062203
**DRD2 pan**	*Dopamine receptor D2*	Rn01418275
**DRD2 short**	*Dopamine receptor D2 short isoform*	Rn01418276
**DRD2 long**	*Dopamine receptor D2 long isoform*	Rn00438543
**DRD3**	*Dopamine receptor D3*	Rn00567568
**DRD4** *	*Dopamine receptor D4*	Rn00564071
**DRD5**	*Dopamine receptor D5*	Rn00562768
**MAOA**	*Monoamine oxidase A*	Rn01430950
**MAOB**	*Monoamine oxidase B*	Rn00566203
**COMT**	*Catechol-O-methyl transferase*	Rn00561037
**VMAT**	*Vesicular monoamine transporter*	Rn00564688
**DAT**	*Dopamine transporter*	Rn00562224

*DRD4 mRNA levels in both regions were too low to be accurately quantitated.

### Statistics

Unless otherwise stated statistical analyses were conducted using SPSS software (IBM SPSS Statistics, version 19) and *p*<0.05 was considered statistically significant. Population outliers were removed by Grubb's test (GraphPad Prism online calculators) on the normalized qPCR, immunoblotting and HPLC data. Immunoblotting data are presented as change of relative intensity compared to the Gdx group ± SEM. Immunoblotting data were normalized to β-actin expression. qPCR data are presented as percent change of mRNA levels relative to the Gdx group ± SEM. Outlier detection of the triplicates obtained from the qPCR raw data excluded measurement errors [42]. qPCR raw data was normalized by the geomean of the housekeepers. HPLC data is expressed as ng/mg tissue ± SEM. HPLC raw data was normalized to an internal control run each day. Dopamine turnover was calculated before outliers were removed. All data was analyzed by one-way ANOVA followed by Fisher's LSD. Comparisons of DAT protein levels were made using one-directional t tests (GraphPad Prism) due to an *a priori* hypothesis [43], based on mRNA findings, that DAT protein would be increased by androgens relative to the Intact and Gdx groups.

## Results

### Circulating sex steroids over a two week adolescent period change mRNAs encoding for pre-synaptic dopamine reuptake, vesicular packaging and dopamine receptor proteins in the substantia nigra

**Figure 1:**
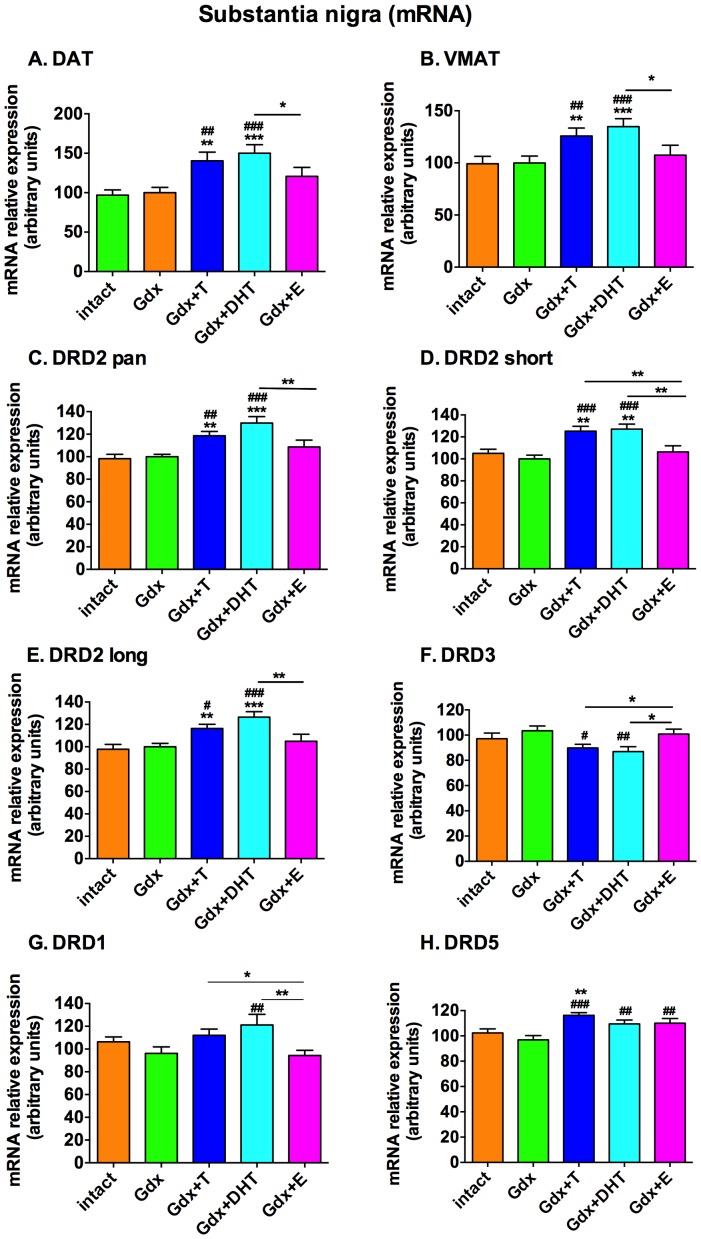
Effect of gonadectomy and sex steroid replacement on presynaptic dopamine transporter and dopamine receptor mRNA expression in the substantia nigra of adolescent male rats. DAT **(A)** and VMAT **(B)** mRNA expression were increased by androgens but not by 17β-estradiol replacement. **(C)** DRD2 pan, **(D)** DRD2 short and **(E)** DRD2 long mRNAs were increased by testosterone and DHT replacement relative to Intact and Gdx groups. 17β-estradiol replacement had no effect on DRD2pan, D2S or D2L mRNA levels. **(F)** DRD3 mRNA was decreased by testosterone and DHT replacement relative to Gdx. 17β-estradiol replacement had no effect on DRD3 mRNA. **(G)** DRD1 mRNA was increased by DHT replacement relative to Gdx. Testosterone and 17β- estradiol replacement had no effect on DRD1 mRNA expression. **(H)** DRD5 mRNA was increased by testosterone replacement relative to Intact and Gdx groups and increased by DHT and 17β-estradiol replacement relative to the Gdx group. * denotes comparison with Intact, unless comparison is indicated by a line, # denotes comparison with the Gdx group, * and # *p*<0.05, ** and ## *p*<0.01, *** and ### *p*<0.001, n = 12–15/group.

In the substantia nigra, there was a significant effect of treatment group on DAT mRNA (F = 6.1, df =  (4,64), *p*<0.0001) (Fig. 1A) and VMAT mRNA (F = 4.18, df  =  (4,65), *p* = 0.005) (Fig. 1B). Both DAT and VMAT mRNAs were increased significantly relative to Intact or Gdx by replacement with T or DHT but not E. DAT mRNA was increased 40% and 50% by T and DHT, respectively, when compared to the Gdx group and 45% and 54% by T and DHT, respectively, when compared to the Intact group. VMAT mRNA was increased 26% and 35% by T and DHT, respectively, when compared to the Gdx group and 27% and 36% by T and DHT, respectively, when compared to the Intact group.

In the substantia nigra, we also found a significant effect of treatment group on DRD2 pan mRNA (F = 8.5, df  =  (4,65), *p*<0.0001) (Fig. 1C). DRD2 pan includes two isoforms, DRD2 short (D2S), which has a truncated cytoplasmic loop, and DRD2 long (D2L). There was a significant effect of treatment group on D2S (F = 8.3, df  =  (4,63) *p*<0.0001) and D2L (F = 6.8, df  =  (4,65) *p* = 0.0001) as well as DRD3 (F = 3.38, df  =  (4,61), *p* = 0.015) mRNA expression in the male rats (Fig. 1D, E, F). DRD2 pan, D2S and D2L mRNAs were significantly increased by T and DHT replacement relative to Intact (*p*<0.005) and Gdx (*p*<0.011). E replacement had no effect on any DRD2 mRNA isoform. In contrast, DRD3 mRNA was decreased relative to Gdx by T (*p* = 0.012) and by DHT replacement (*p* = 0.004) but was unchanged by E replacement.

We found a significant effect of treatment group on DRD1 and DRD5 mRNA expression in the substantia nigra (F = 3.3, df  =  (4,62), *p* = 0.017 and F = 5.9, df  =  (4,61), *p* = 0.0005, respectively) (Fig. 1G, H). DHT replacement significantly increased DRD1 mRNA relative to Gdx (*p* = 0.006). There was only a trend for T to increase DRD1 mRNA expression relative to Gdx (*p* = 0.064) and E replacement had no effect on DRD1 mRNA. Both T and DHT groups had significantly increased DRD1 mRNA relative to the E group (*p* = 0.04 and 0.004, respectively). In terms of DRD5 mRNA, T replacement significantly increased transcript levels relative to Intact (*p* = 0.003) and Gdx (*p*<0.001). Both DHT replacement and E replacement significantly increased DRD5 mRNA relative to Gdx (Gdx vs. Gdx+DHT, *p* = 0.007; Gdx vs. Gdx+E, *p* = 0.003).

### Circulating sex steroids over a two week adolescent period do not change levels of mRNAs encoding for proteins involved in dopamine breakdown, but sex steroids do change dopamine receptor mRNA levels in the dorsal striatum

**Figure 2:**
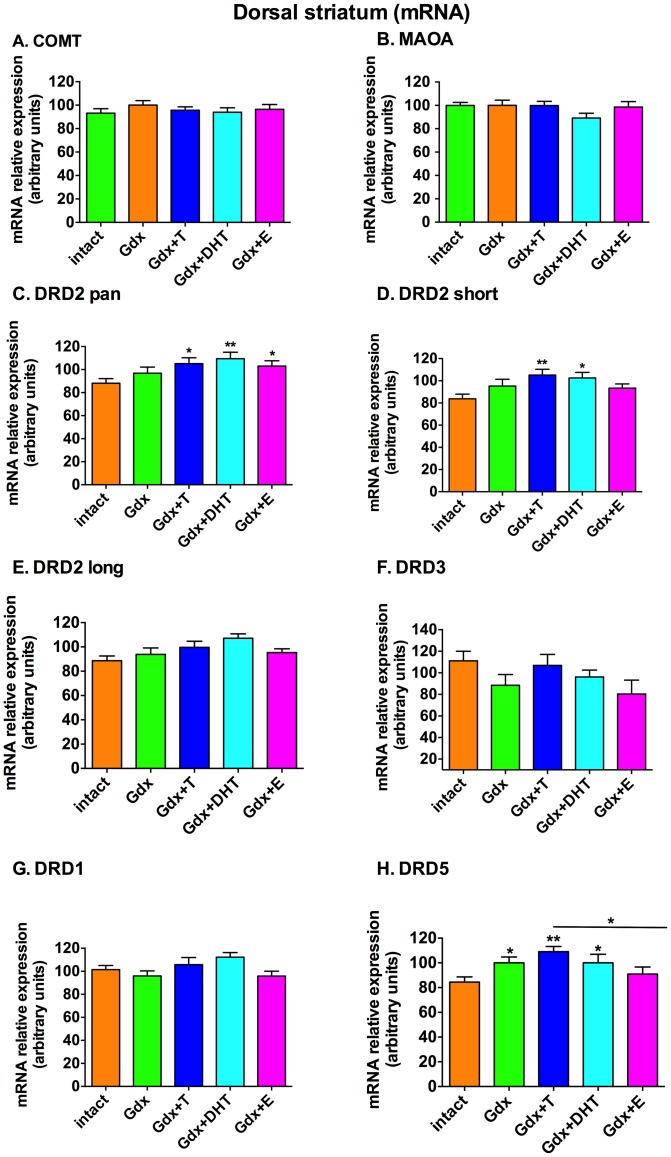
Effect of gonadectomy and sex steroid replacement on dopamine metabolic enzyme and dopamine receptor mRNA expression in the dorsal striatum of adolescent male rats. There was no effect of treatment group on mRNA expression of COMT **(A)** or MAOA **(B)**. **(C)** DRD2 pan mRNA was increased by testosterone, DHT and 17β-estradiol replacement relative to the Intact group. **(D)** DRD2 short mRNA was increased relative to the Intact group by testosterone and DHT replacement but not by17β-estradiol replacement. **(E)** There was a trend for treatment group to change DRD2 long mRNA expression (F =  2.36, df  =  (4,65), *p* = 0.063). There were no statistically significant sex steroid-related changes in **(F)** DRD3 mRNA (F = 1.62, df  =  (4,65), *p* = 0.18) or **(G)** DRD1 mRNA (F = 2.10, df  =  (4,65), *p* = 0.015) **(H)** DRD5 mRNA expression was increased relative to the Intact group by Gdx, testosterone and DHT replacement but not by 17β-estradiol replacement. * denotes comparison with intact, * *p*<0.05, ** *p*<0.01, n = 12–16/group.

In the striatum, COMT, MAOA and MAOB mRNAs were unchanged by gonadectomy and sex steroid replacement [COMT, F = 1.218, df  =  (4,66), *p* = 0.311 (Fig. 2A); MAOA, F = 1.36, df  =  (4,69), *p* = 0.257 (Fig. 2B); MAOB, F = 0.787, df  =  (4,68), *p* = 0.538 (data not shown)].

In the striatum, we also found a significant effect of treatment group on DRD2 pan mRNA (F = 2.72, df  =  (4,66) *p* = 0.036). DRD2 pan mRNA was increased by T, DHT and E relative to the Intact group (Fig. 2C). The significant increase in DRD2 pan as a result of androgen replacement may be driven by significant increases in DRD2S mRNA (F = 2.85, df  =  (4,67), *p* = 0.03) by T and DHT (Fig. 2D). There were no significant changes in DRD2L mRNA expression due to sex steroid treatment (F = 2.36, df  =  (4,65), *p* = 0.063)(Fig. 2E). There were no statistically significant sex steroid-related changes in striatal DRD3 mRNA (F = 1.62, df  =  (4,65) *p* = 0.18) (Fig. 2F) or DRD1 mRNA (F = 2.10, df  =  (4,65), *p* = 0.091) (Fig. 2G). There was a significant effect of treatment group on DRD5 mRNA expression in the striatum (F = 3.34, df  =  (4,65), *p* = 0.015) (Fig. 2H). DRD5 mRNA expression was increased by gonadectomy alone relative to Intact (Gdx vs Intact, *p* = 0.035) and this increase was not attenuated by the replacement of T or DHT (Intact vs Gdx+T, *p* = 0.001, Intact vs. Gdx+DHT, *p* = 0.048) but was prevented by E replacement (Intact vs Gdx+E, ns)(Fig. 2H).

### Dopamine transporter protein is increased by testosterone in the substantia nigra

**Figure 3:**
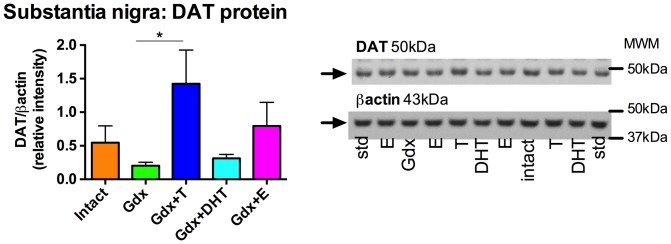
Effect of gonadectomy and sex steroid replacement on dopamine transporter protein in the substantia nigra of adolescent male rats. In the substantia nigra there was a trend for treatment group to change DAT protein (F = 2.52, df  =  (4,41), *p* = 0.056). Representative immunoblots of a subset of substantia nigra samples show DAT (50 kDa) and β-actin (43 kDa). *A priori*, planned comparisons, based on mRNA data, revealed that testosterone replacement increased DAT protein compared to the gonadectomised group. * *p*<0.05, n = 9–10/group. (MWM, molecular weight marker; std, internal standard).

In the substantia nigra, we found that sex steroid effects on DAT protein reached trend levels of statistical significance overall (F = 2.52, df  =  (4,41), *p* = 0.056) (Fig 3). *A priori*, planned comparisons revealed significant increases in DAT protein in response to testosterone (Gdx vs. Gdx+T, *p* = 0.024, t = 2.15, df  = 16) but not in response to DHT (Gdx vs. Gdx+DHT, *p* = 0.09, t = 1.36, df  = 16) (Fig. 3). We did not detect a significant increase in DAT protein levels in the testosterone or DHT groups compared to the Intact group (Intact vs. Gdx+T, *p* = 0.075, t = 1.51, df  = 17; Intact vs. Gdx+DHT, *p* = 0.16, t = 0.96, df  = 17). We have previously reported testosterone-induced increases in TH protein levels in the substantia nigra [**16**]

### Proteins involved in dopamine transport and dopamine synthesis appear unchanged by testosterone in the dorsal striatum

**Figure 4:**
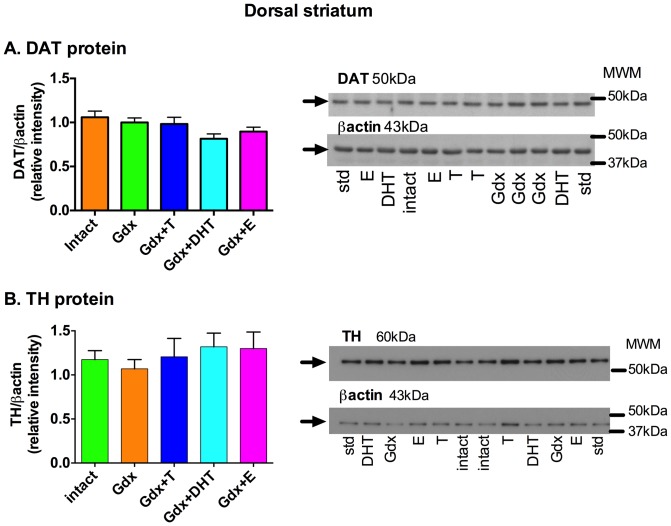
Effect of gonadectomy and sex steroid replacement on dopamine transporter protein and tyrosine hydroxylase protein in adolescent male rat dorsal striatum. **(A)** DAT protein was unchanged in the dorsal striatum by gonadectomy and sex steroid replacement (F = 2.39, df  =  (4,45), *p* = 0.07). Representative immunoblots of a subset of dorsal striatum samples from all five groups show DAT (50 kDa) and β-actin (43 kDa). **(B)** TH protein was unchanged by gonadectomy or sex steroid replacement in the dorsal striatum (F = 0.41, df  =  (4,45), *p* = 0.78). Representative immunoblots of a subset of dorsal striatum samples from all five groups show TH protein (60 kDa) and β-actin protein (43 kDa). * *p*<0.05, n = 9–10/group. (MWM, molecular weight marker; std, internal standard).

There was a trend towards a change in DAT protein levels (normalized to β-actin levels) in the dorsal striatum according to treatment group (F = 2.39, df  =  (4,45) *p* = 0.07). However, *a priori*, planned comparisons revealed no effect of testosterone or DHT relative to Intact or Gdx groups on DAT protein levels in the dorsal striatum (all *p*>0.05, Fig. 4A).

Striatal TH protein levels (normalized to β-actin levels) were also unchanged by gonadectomy or by sex steroid replacement in the dorsal striatum (F = 0.41, df  =  (4,45), *p* = 0.78) (Fig. 4B).

### Dopamine turnover in the dorsal striatum is increased by testosterone removal

**Figure 5:**
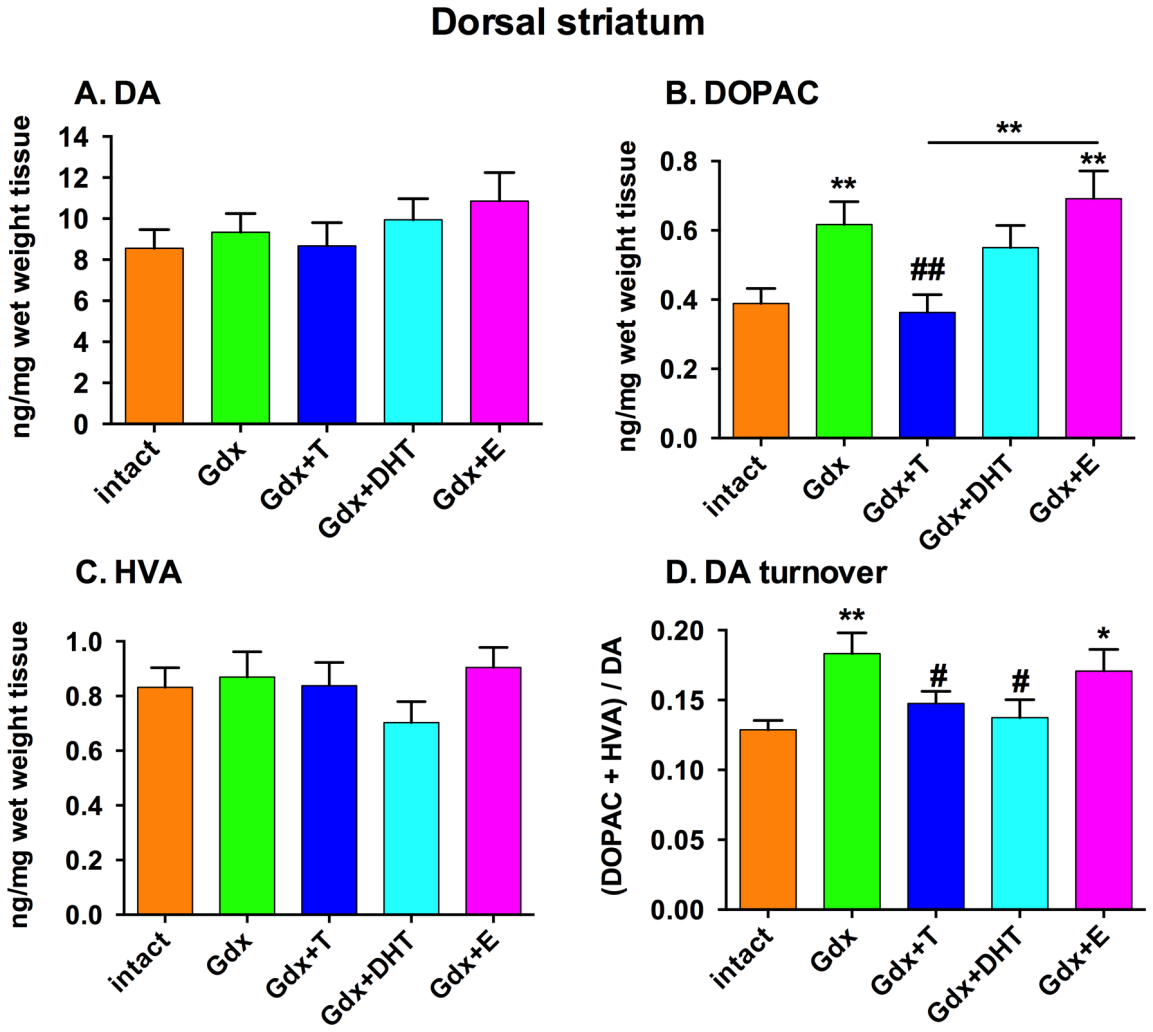
The effect of gonadectomy and sex steroid replacement on dopamine, dopamine metabolites and dopamine turnover in the dorsal striatum of adolescent male rats. There was no significant effect of treatment group on dopamine **(A)** or HVA **(C)**. Gonadectomy increased DOPAC **(B)** and this increase was attenuated by testosterone replacement only. Dopamine turnover **(D)** was significantly increased by gonadectomy and this was attenuated by testosterone and DHT replacement but not 17β-estradiol replacement. * denotes comparison with Intact unless comparison indicated by a line, # denotes comparison with the Gdx group, * and # *p*<0.05; ** and ## *p*<0.01, n = 11–16/group for all.

Dopamine and HVA levels were unchanged in the striatum in any treatment group (F = 0.74, df  =  (4,67), *p* = 0.57 and F = 0.8, df  =  (4,61), *p* = 0.53, respectively) (Fig. 5A, C). There was a significant effect of treatment group on levels of the DOPAC in the striatum (F = 5.3, df  =  (4,68), p = 0.001). Striatal DOPAC was increased in the Gdx group compared to the Intact group (*p* = 0.004) and this was prevented by testosterone replacement (Gdx vs. Gdx+T, *p* = 0.003), but not by DHT or E (Fig. 5B). The increase in DOPAC in the Gdx group resulted in a significant group effect on dopamine turnover (DOPAC+HVA/DA) (F = 3.2, df  =  (4,64), *p* = 0.019; n = 11–16) (Fig. 5D). Dopamine turnover was increased in the Gdx group (Intact vs. Gdx, *p* = 0.005) and the increase was prevented by androgens (Gdx vs T, *p* = 0.04; Gdx vs. DHT, *p* = 0.012), but not by E (Gdx vs. Gdx+E, ns) (Fig. 5D).

## Discussion

### Summary of findings

We provide data to support our hypothesis that testosterone may modulate, via mainly androgen receptor-driven changes, gene expression of multiple molecules involved in the regulation of dopamine within the nigrostriatal pathway in adolescent male rat brain. In the substantia nigra, testosterone increased several dopamine receptor mRNAs [D1, D2 (short and long), D5] as well as dopamine transporter (VMAT2, DAT) gene expression and DAT protein but decreased DRD3 mRNA. This novel data, combined with our previous findings of increased TH protein and COMT and MOA mRNAs in the substantia nigra [16] with testosterone during an adolescent period (PND45-60), suggests that the dopaminergic system may be coordinately modified to accommodate more midbrain dopamine signaling when males mature. Adolescent sex steroids also regulated both D2 and D5 dopamine receptor mRNAs in the dorsal striatum (summarized in Table 2). Our data highlight in adolescent male rats that 1) there are several different points at which testosterone can modulate molecular indices of dopamine signaling, 2) there is a greater degree of testosterone regulation of dopamine-related molecular parameters in the substantia nigra compared to the striatum and 3) testosterone induces a coordinated increase in DRD2S mRNA levels in the substantia nigra and striatum.

**Table 2:** Summary of gene expression and protein changes in response to sex steroid replacement relative to the Gdx group.

	Substantia nigra			Dorsal striatum		
	T	DHT	E	T	DHT	E
**mRNA**						
**DAT**	+++	+++	=	na	na	na
**VMAT**	++	+++	=	na	na	na
**DRD1**	=	++	=	=	=	=
**DRD2 pan**	+	+++	=	=	=	=
**DRD2S**	++	++	=	=	=	=
**DRD2L**	+	++	=	=	=	=
**DRD3**	-	-	=	=	=	=
**DRD4**	nd	nd	nd	nd	nd	nd
**DRD5**	++	+	+	=	=	= ?
**TH**	= ^$^	= ^$^	= ^$^	na	na	na
**COMT**	+ ^$^	+ ^$^	= ^$^	=	=	=
**MAOA**	+ ^$^	++ ^$^	= ^$^	=	=	=
**MAOB**	+ ^$^	+ ^$^	= ^$^	=	=	=
**protein**						
**DAT**	+++	=	=	=	=	=
**TH^$^**	+++ ^$^	= ^$^	= ^$^	=	=	=

nd, not determined; na, not applicable;  = , no change; +, <20% increase; ++, 20–30% increase; +++, >30% increase; -, <20% decrease; $, previously reported data [16].

### Testosterone has differential effects on dopamine in the substantia nigra versus striatum

Dopamine production is rate limited by TH levels and TH activity. Previous studies in rodent brain have reported both increases and decreases in striatal dopamine in response to sex steroids [19]–[21], [44]. We have reported that testosterone replacement increased TH protein levels in the substantia nigra in adolescent male rats [16], however, we now show that contrary to our prediction that testosterone would increase TH in the striatum as well, no increase in striatal TH protein or increase in striatal dopamine levels was detected. In fact, dopamine turnover (a measure of dopamine activity) in the striatum was increased upon testosterone removal and this stimulatory effect was attenuated by testosterone replacement. The somewhat surprising increase in dopamine turnover in the striatum following gonadectomy could reflect an increase in dopamine packaging and release that is maintained in balance by dopamine reuptake and breakdown. However, when comparing gonadectomised and intact rats, changes in dopamine breakdown enzyme or transporter mRNAs or proteins (where measured) were not found in the striatum, thus the changes in dopamine turnover after gonadectomy may reflect post-transcriptional or post-translational changes in the activity of these proteins.

### Testosterone may regulate dopamine neurotransmission at dopaminergic cell soma and terminals

Gene expression changes in dopaminergic cell bodies can alter protein levels in both presynaptic axon terminals at a distance from the cell bodies and/or locally in somatodendritic fields. Our previously reported increase in TH protein in the region of the dopaminergic cell bodies [16] combined with the lack of increase in TH protein or dopamine in the terminals in the striatum suggests that increases in dopamine synthesis via androgens may occur at the level of cell bodies and dendrites rather than at the terminals. In support of the preference for testosterone to induce local changes in molecular indices of dopamine signaling, we find that increases in DAT protein are found proximal to the soma, in the substantia nigra and not distal, in the striatum. Increased dendritic synthesis, release and uptake of dopamine could serve to modify feedback inhibition of dopamine neurons within the substantia nigra, thereby regulating dopamine neuron excitability and indirectly modifying dopamine release at axon terminals [45], [46]. The fact that localized changes in molecules impacting local self-regulation of dopamine neurons may be more directly influenced by testosterone could have relevance for schizophrenia as a recent meta-analysis of imaging studies concluded that the largest dopaminergic abnormality in schizophrenia is presynaptic [4], which implicates an intrinsic dysfunction in dopamine neurons themselves. DAT [47]–[49] and VMAT [50] have been localized to both the somatodendritic field and terminals of nigrostriatal dopaminergic neurons in the rat and human. Our reported increase in VMAT and DAT mRNAs in the substantia nigra in response to testosterone could lead to increased DAT and VMAT protein in either the dendrites and/or in the terminals and our data (increased DAT protein only in the region of cell bodies not at the terminals) supports that dopamine neuron dendrites are more likely targets of testosterone-induced changes. Testosterone-induced increased somatodendritic dopamine transport and dopamine breakdown [16] in the substantia nigra would serve to not only maintain dopaminergic homeostasis but also provide more precise temporal control over the activity of the dopaminergic neuron cell bodies. Further, the increase in DAT and VMAT mRNAs by testosterone was not accompanied by changes in TH protein or dopamine levels in the striatum perhaps suggesting that synthesis and steady state levels are stable at the terminals, whereas in the substantia nigra both TH and DAT protein are increased by testosterone. Thus, our data suggest that dopamine neurotransmission may be enhanced at the cell bodies and dendrites of dopamine neurons in response to testosterone at male adolescence. Ultimately, the functional outcome of these molecular changes would be determined by the balance between theses changes e.g. increased DRD2 in the somatodendritic field (see below) would increase feedback inhibition of the DA neurons, but increased DAT would serve to clear DA from the extracellular space more rapidly, thereby potentially reducing feedback inhibition.

### Testosterone regulates the level of dopamine receptor mRNAs in the nigrostriatal pathway

In support of a local change proximal to dopamine neurons in the substantia nigra, we find that 3 out of 5 dopamine receptor mRNAs are increased in response to adolescent testosterone in the substantia nigra perhaps reflecting changes in available dopamine. We also report increased DRD2 gene expression in response to testosterone in both the region of the dopaminergic cell bodies and in the region of the medium spiny neurons of the dorsal striatum. As such, there are multiple sites at which increases in DRD2 expression could modulate dopamine sensitivity of the nigrostriatal pathway: at the cell bodies and dendrites of dopamine neurons within the midbrain, at the pre-synaptic dopamine terminals in the striatum and at the post-synaptic neurons in the striatum. DRD2 activation at the dopaminergic cell bodies results in the attenuation of dopaminergic neuron excitability via feedback inhibition. DRD2 and DRD3 are considered autoreceptors in dopaminergic neurons where DRD2 at the presynaptic terminal provides inhibitory control over dopamine release and DRD2 and DRD3 control electrical activity of the dopamine neurons at the cell body. The testosterone-induced changes we report in DRD2 gene expression in the nigrostriatal pathway at adolescence are of particular interest as DRD2 receptors are the target of all antipsychotic treatments for schizophrenia, with DRD2 receptors in the dorsal striatum suggested to be the most responsive to changes in tissue dopamine levels [7]. Our evidence indicates that testosterone also increases DRD1 and DRD5 (excitatory receptors) in the substantia nigra and DRD5 in the striatum, suggesting more generalized sensitivity to secreted dopamine via testosterone exposure. Interestingly, gonadectomy also increased DRD5 mRNA in the striatum and increased DRD5 mRNA was attenuated by estradiol and not androgens and this effect of estrogen suggests that the balance of sex steroids may play a role in the regulation of striatal DRD5 gene expression. It is important to acknowledge that DHT can also have effects via conversion to 3β-diol, which has a high affinity for ERβ and DHT effects may therefore include an estrogenic component [11]. In conclusion, the action of dopamine would, in combination with DA synthesis, transport and metabolism, depend on the balance of excitatory and inhibitory dopamine receptors and their location within the nigrostriatal pathway and the gene expression profile of these DA-related molecules can be modulated by sex steroids in male adolescence.

### The mechanism of testosterone regulation of molecular indices of dopamine neurotransmission is mostly androgenic

Crucial to developing new drug targets for therapy in dopamine-related neural disorders is knowledge of the underlying mechanism(s) driving the changes in dopamine regulating proteins. The majority of the gene expression changes reported here are only induced by DHT and testosterone, and not by estradiol, indicating that in adolescent males androgen receptor, not ERα, activation is critical for these responses. It is less clear whether ERβ is involved or not as DHT, via conversion to 3β-diol has a high affinity for ERβ [11]. The minimal estrogenic effects driven by testosterone may provide some insight in to the greater sensitivity of males to schizophrenia [51]–[53] and to dopamine-potentiating drugs of abuse such as amphetamine, which, in males, could potentially be due to a combination of a lower level of protective estrogen-driven effects [2] as well as potentiating dominant androgen-driven effects. Compounding these AR-driven effects is our evidence that in addition to modulating dopamine regulating proteins and mRNAs, testosterone also creates a self-reinforcing, positive feedback loop in the adolescent male rat midbrain to create a more androgen responsive state by increasing AR gene expression whilst decreasing ERα gene expression [16].

### Limitations of our study

It is important to note the lack of detectable differences in gene expression between intact and gonadectomised rats. Differences in dopamine pathway-related gene expression between intact and gonadectomised rats in our study may be subtle and difficult to detect due to differences between the natural gradual increase in testosterone over adolescence and the immediate, supraphysiological, steady state levels achieved with implants. Intact animals may require a longer exposure to high testosterone to allow the changes we see in gene expression, that we report in sex steroid replaced animals, to occur. The lack of detectable differences between intact and gonadectomised rats could occur if gonadectomy had occurred after the adolescent increase in testosterone. Although androgen-dependent physiological changes (preputial separation) at adolescence are reported in male Sprague-Dawley rats as early as day postnatal day 38 [54] they are more commonly reported between postnatal days 45 and 48 [55]. The majority of studies agree, however, that testosterone levels continue to rise between 45 to 60 days of age [30]–[34], and measurements of circulating testosterone in our Sprague-Dawley rat colony is in agreement with this (unpublished data). In addition, the male brain is not testosterone naïve prior to when testosterone levels start to increase [31]. We also demonstrate here differences in striatal dopamine turnover between Intact and Gdx animals as well as significant differences in seminal vesicle weights [16] and in BDNF-related pathways in the CNS (unpublished data), all of which support the efficacy of our paradigm. We acknowledge that the serum sex steroid levels achieved via implants are supraphysiological, however our data indicate that molecular indices of dopamine neurotransmission in the adolescent male nigrostriatal pathway can be modulated by sex steroids and the replacement studies allow us to dissociate androgen versus estrogen driven mechanisms of testosterone, both important for sex steroid based drug development. The duration of treatment may also account for at least some of the conflicting data in the literature. Changes reported in the adult rat prefrontal cortex indicate decreased dopamine concentrations four days post gonadectomy but dopamine was increased at 28-days post gonadectomy [56]. We detected no change in dopamine concentration in the dorsal striatum at 14 days after gonadectomy but an increase in dopamine turnover in response to gonadectomy – thus, our data may reflect a transitionary phase between 4 and 28 days of replacement. We did not determine how these dopamine-related changes may vary over time.

A limitation of this study is that we do not know exactly how the dopamine receptor mRNAs are translated into protein as there are different synthetic pools of dopamine receptor mRNA (striatal medium spiny neurons, dopamine neurons) and protein measurements of the receptors in the striatum would reflect a mixture of receptors from different mRNA pools and would not distinguish the source or the cellular location (post-synaptic versus pre-synaptic) of any protein changes in the striatum, making conclusions about protein expression in the dorsal striatum difficult to interpret. However, gene expression data provides valuable information about differential control of these molecules at the mRNA level at different points in the nigrostriatal pathway. In contrast, protein measurements of DAT and TH are more informative as the primary source of these proteins is the dopamine neurons in the nigra, allowing conclusions to be drawn regarding changes in protein expression in the somatodendritic and terminal fields of the nigral neurons. An important follow up to this study will be to determine whether these observed gene expression and protein changes in dopamine-related molecules lead to changes in behavior in response to dopamine-potentiating drugs such as amphetamine.

### Conclusions

In general, our data support the hypothesis that the ability of the nigrostriatal pathway to respond to dopamine is modulated by circulating testosterone levels during adolescence. Although the current data does not allow us to draw conclusions regarding the functional outcomes of observed changes it is feasible that in individuals with an underlying susceptibility to schizophrenia the pubertal increase in circulating testosterone at adolescence may serve as a trigger for the presentation of dopamine-related psychosis. Studies in humans suggest that increased testosterone increases striatal dopamine. Studies in women using functional magnetic resonance imaging support the hypothesis that exogenous testosterone affects dopaminergic activity [57] and our study, albeit in males, indicates the potential molecular correlates that may underlie this. Susceptibility to schizophrenia may relate to variations in dopamine-regulating genes or variation in sex steroid-related genes. Indeed, genetic variation in a number of genes involved in dopamine regulation have been linked to schizophrenia risk, including DRD2 [58], [59], VMAT2 (psychotic disorder) [60] and COMT [61], [62]. A variant of the ESR1 gene has also been shown as a genetic risk factor for schizophrenia [63]. In the current work, we have not investigated the functional effects of these changes on brain dopamine but we note that human studies report that increased testosterone increases striatal dopamine [57] and anabolic androgenic steroids, synthetic variants of testosterone, are known to be associated with adverse effects on mental health, including psychosis and aggression [64]. Our study demonstrates changes that may represent the molecular correlates of such effects.

We conclude that the testosterone-induced, AR-driven modulation of molecular indices of dopamine responsivity of the nigrostriatal pathway may involve regulation of dopamine feedback inhibition in the somatodendritic field and post-synaptic dopamine action in the terminal field. Testosterone has widespread effects including regulating mRNA and some protein levels of molecules involved in pre-synaptic dopamine synthesis, dopamine reuptake and dopamine packaging, dopamine breakdown and dopamine reception. Although the functional outcome of these molecular changes have not been measured this knowledge provides clues to the coordinated multi-faceted level of sex steroid control over dopamine neurotransmission to target for drug development, whereby dopamine responsivity of the nigrostriatal pathway can be modulated, perhaps by changing androgen and/or estrogen receptor activation.
